# Local and regional flaps in scalp reconstructions: A retrospective analysis

**DOI:** 10.1016/j.jpra.2025.03.005

**Published:** 2025-03-14

**Authors:** Adam Stepniewski, Julian Daugardt, Alperen Sabri Bingoel, Jessica Hoffmann, Katharina Jäckle, Tomasz Korzeniowski, Philipp Kauffmann, Wolfgang Lehmann, Gunther Felmerer

**Affiliations:** a Division of Plastic Surgery, Department for Trauma Surgery, Orthopedics and Plastic Surgery, University Medical Center Göttingen, Göttingen, Germany; b Department of Traumatology, Hand Surgery and Orthopedics, Klinikum Bremerhaven Reinkenheide, Bremerhaven, Germany; c Department for Trauma Surgery, Orthopedics and Plastic Surgery, University Medical Center Göttingen, Göttingen, Germany; d Department of Plastic, Reconstructive Surgery and Burn Treatment, Faculty of Medicine, Medical University of Lublin, Leczna, Poland; e Department of Oral and Maxillofacial Surgery, University Medical Center Göttingen, Göttingen, Germany

**Keywords:** Local flap, Regional flap, Fasciocutaneous flap, Scalp defect, Reconstruction

## Abstract

The study demonstrates the broad applicability of local and regional flaps for the treatment of scalp defects of varying size and etiology. We aimed to verify whether the following variables correlate with the postoperative complication rate, length of hospitalization, and postoperative inpatient course, i.e., etiology of the defect, surgical technique, duration of surgery, defect size, localization, comorbidities, and neoadjuvant radiation. We examined whether the complications had an impact on the hospitalization and postoperative inpatient course. The inclusion criteria were defects in the scalp, reconstruction between 2010 and 2021, and use of local or regional fasciocutaneous flap. In this study, the following parameters were considered such as gender, age, main diagnosis, comorbidities, defect etiology, size and localization, type of surgical treatment, surgery duration, hospitalization, postoperative inpatient course, local complications, and revisions. A total of 39 patients (mean age: 56.5 ± 20.5 years) were selected. Postoperative complications did not correlate with defect etiology, surgical technique, defect size, defect localization, surgery duration, comorbidities, and local radiation. Postoperative complications, comorbidities, and neoadjuvant radiotherapy influenced the length of hospitalization. The postoperative inpatient course was influenced by the surgery duration and postoperative complications. Defect etiology, surgical technique, defect size, and defect localization had no impact on the overall hospitalization. Local and regional flaps are safe and reliable options for the treatment of scalp defects up to 80 cm². Their use in the study resulted in excellent functional and cosmetic outcomes. The risk of complications and revisions is considered as low.

## Introduction

Defects in the scalp are not just a cosmetic problem and they can have diverse etiologies. In terms of therapy, various methods are available according to the reconstructive supermarket model.^1^^,^^2^ However, patient-specific factors must be considered in the therapy, e.g., comorbidities, age, previous chemotherapy or radiotherapy, and previous operations with scarring, all of which increase the likelihood of postoperative local complications and may require revision.^3^

The aim of this study was to demonstrate the broad applicability of local and regional flaps for the treatment of scalp defects of varying size and origin.

Furthermore, we wanted to investigate whether the following variables correlated with the postoperative local complication rate, length of hospitalization (HS), and postoperative inpatient course (POC), i.e., the etiology of the defect, surgical technique, duration of surgery (DOS), defect size, defect localization, comorbidities, and neoadjuvant radiation. We also investigated whether the complications influenced HS and POC.

## Material and methods

The inclusion criteria for the retrospective, anonymized cohort study were scalp defect and reconstruction between 2010 and 2021 with local or regional fasciocutaneous flap. Flap designs that were not precisely documented were referred to as “other local flaps.” If the defect size was not well documented, the intraoperative photographs were used to determine the size. Rulers or objects in the images, such as surgical instruments, were used as a reference. If defect sizes could not be determined definitively, these cases were included in some of the statistics due to their clinical importance.

The following parameters were considered: gender, age, main diagnosis, comorbidities, defect etiology, size and localization, type of surgical treatment, DOS, neoadjuvant radiotherapy, HS, POC, local complications, and revisions.

The statistical analysis was performed using IBM^Ⓡ^ SPSS^Ⓡ^ Statistics Version 27.0.1.0. The significance level was set at a p-value of 0.05.

The study was approved by the Ethics Committee of the University Medical Center Göttingen on 08.04.2022 with reference number 14/8/22.

## Results

The cohort consisted of 39 patients (f:m = 25:14). Mean age was 56.5 ± 20.5 years (16–90 years), 54.5 ± 19.8 years for women and 60.1 ± 21.9 years for men. HS was 18.7 ± 14.2 days (3–67 days), POC 13.1 ± 9.2 days (2–49 days). Nine patients (23%) developed local complications, 4 (10.2%) required revision.

### Defect etiology

Seven cases underwent reconstruction due to trauma, 5 due to skin tumor excision, and 27 due to impaired wound healing. The defects were successfully closed in all patients by the time they were discharged.

### Trauma

This group consisted of 7 patients (f:m = 3:4). The mean age was 32.1 ± 20 years (16–62 years). Two of the 7 defect sizes could be determined. The average defect size was 12.5 cm² ± 16 cm² (1.2–23.8 cm²), HS was 13.3 ± 14.4 days (3–45 days), and POC was 10.7 ± 10.9 days (2–34 days). Local epitheliolysis in 2 patients healed spontaneously.

### Skin tumor excision

Five defects (f:m = 3:2) resulted from skin tumor excision. The mean age was 77.2 ± 5.4 years (69–83 years), average defect size was 23.2 cm² ± 25.6 cm² (3.1–67.9 cm²), HS was 19.6 ± 14.2 days (3–37 days), POC was 12.2 ± 10.2 days (2–29 days). One case of wound dehiscence was revised.

### Impaired wound healing

This group consisted of 27 cases (f:m =19:8) with the mean age of 59 ± 16.4 years (36–90 years). Among the 27 defect sizes, 19 could be determined. The mean defect size was 23.2 cm² ± 25.6 cm² (1.8–78.5 cm²), HS was 20 ± 14.4 days (8–76 days), POC was 13.9 ± 8.8 days (7–49 days). Five wound dehiscences and 1 case of skin necrosis were observed, 3 of which required revision.

### Surgical technique

*Rotation flaps* were used in 16 patients (f:m = 12:4) with a mean age of 59.4 ± 19.7 years (20–90 years). Among the 16 defect sizes, 10 could be determined. The average size was 25.7 cm² ± 21.3 cm² (3.1–67.9 cm²), HS was 23 ± 16.6 days (8–67 days), POC was 14 ± 8.1 days (6–34 days). Complications occurred in 5 cases, 3 of which required revision.

*Advancement flaps* were used in 5 patients (f:m = 3:2) with mean age of 54 ± 18.9 years (22–71 years). Three of the 5 defect sizes could be determined. The average defect size was 8 ± 5.2 cm² (4.2–14 cm²), HS was 24.6 ± 20.2 days (7–56 days), POC was 17.4 ± 17.8 days (6–49 days). One wound dehiscence required revision.

Seven patients (f:m = 5:2) were treated using *regional flaps*. The mean age was 43.3 ± 20.5 years (16–69 years). Five of the 7 defect sizes could be determined. The average defect size was 22.5 ± 28.1 cm² (1.2–56.2 cm²), HS was 24.6 ± 20.2 days (7–56 days), POC 8.1 ± 4 days (2–14 days). One case of wound healing disorder and 1 case of epitheliolysis were healed by secondary intention.

In 11 cases (f:m = 5:6), *other local flaps* were used. The average age was 61.7 ± 21.1 years (27–83 years). Among the 11 defects, 8 could be determined. The mean size was 34.1 ± 26.4 cm² (7.8–78.5 cm²), HS was 16 ± 8.4 days (3–29 days), POC was 13.1 ± 7.4 days (2–28 days). One wound atrophy was observed, no revision was necessary.

### Defect size

Among the 39 cases, defect sizes could not be determined in 13, which were not included in the following calculations.

The mean defect size was 25.6 ± 23.4 cm² (1.2–78.5 cm²). We divided the defect sizes into 2 small (<2 cm²), 15 medium-sized (2–25 cm²), and 9 large (>25 cm²) defects.

The area of the small defects was 1.5 ± 0.4 cm² (1.2–1.8 cm²), HS was 11.5 ± 4.9 days (8–15 days), POC was 10.5 ± 4.9 days (7–14 days). One epitheliolysis healed spontaneously.

The area of the medium-sized defects was 11.4 ± 6.4 cm² (3.1–23.8 cm²), HS was 19.6 ± 15.8 days (3–67 days), POC was 11.6 ± 5.5 days (2–24 days). One of the 2 wound dehiscences was revised.

The area of the large defects was 54.6 ± 13.4 cm² (37.8–78.5 cm²), HS was 18.9 ± 9.5 days (9–37 days), POC was 15 ± 8.1 days (8–29 days). Four minor complications were documented, none required revision.

### Defect localization

The defects were not evenly distributed over the areas of the scalp: 21 were frontal, 5 were temporal, 4 were parietal, 3 were temporo-parietal, and 1 lesion each were frontotemporal, frontoparietal, parietooccipital, occipital, occipitotemporal, and retroauricular.

### Duration of surgery

The mean DOS was 125.4 ± 54.9 min (38–276 min).

### Complications

Local complications occurred in 9 cases, 4 of which were revised. Patients with complications had DOS 130.8 ± 64.0 min (60–276 min), HS was 29 ± 21.9 days (7–67 days), and POC was 20.2 ± 14.1 days (6–49 days), and in patients without complications DOS was 121.6 ± 52.8 min (38–214 min), HS was 14.6 ± 8.3 days (3–33 days), and POC was 10.1 ± 4.6 days (2–24 days).

### Comorbidities

Comorbidities were documented in 29 patient: 17 had metabolic diseases (diabetes mellitus type II, hypothyroidism, and obesity), 12 had neurological diseases (epilepsy and polyneuropathy), 15 had cardiovascular diseases (arterial hypertension and atrial fibrillation), 5 had addictions (nicotine abuse and drug abuse), and 1 case had infectious diseases (HIV and chronic hepatitis C).

HS for patients without comorbidities was 10.6 ± 4.1 days (3–16 days), with 1 comorbidity it was 28.6 ± 17.4 days (11–67 days), and with several comorbidities HS was 16.4 ± 10.1 days (3–37 days); POC was 8.6 ± 3.8 days (2–12 days), 19.9 ± 11.4 days (8–49 days), and 10.5 ± 5.6 days (2–29 days), respectively.

### Preoperative radiotherapy

Among the 39 patients, 13 received radiotherapy (f:m = 10:3). Their mean age was 75 ± 15.1 years (39–83 years), HS was 25 ± 11.5 days (9–67 days), POC was 13.7 ± 6.5 days (7–29 days). Three cases developed local complications, 2 of which required revisions.

**Figure 1:**
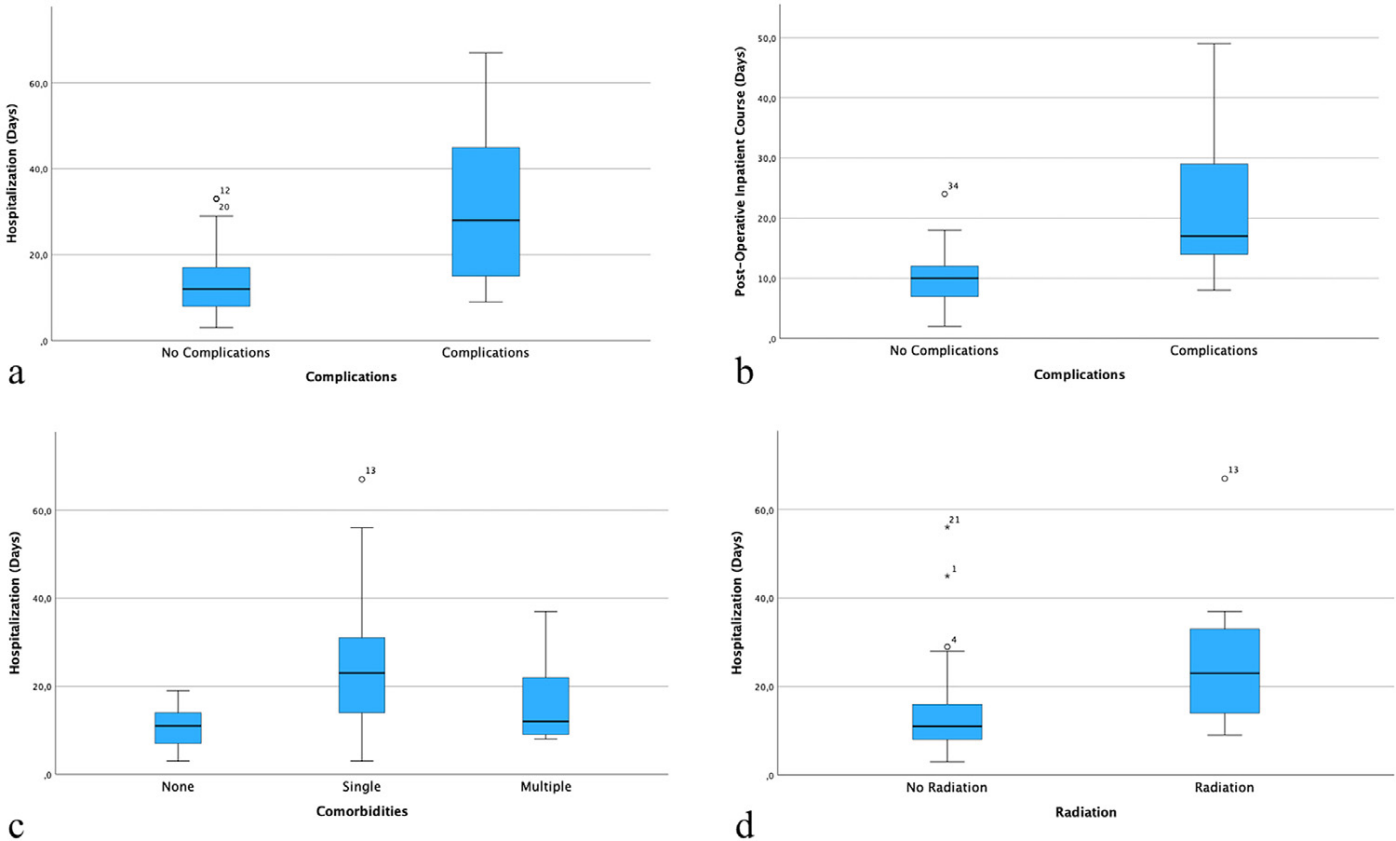
Significant correlations and differences: complications and HS, p = 0.005 (a); complications and POC, p = 0.005 (b); comorbidities and HS, p = 0.022 (c); irradiation and HS, p = 0.006 (d).

A summary of the correlations of the variables with the occurrence of complications and differences in HS and POC are shown in Table 1 and Figure 1.

**Table 1:** Correlations and differences.

	CORRELATION WITH COMPLICATIONS	DIFFERENCE IN HOSPITALIZATION (HS)	DIFFERENCE IN POST-OPERATIVE INPATIENT COURSE (POS)
DEFECT ETIOLOGY	none (p = 0.92)	none (p = 0.08)	none (p = 0.163)
SURGICAL TECHNIQUE	none (p = 0.58)	none (p = 0.08)	none (p = 0.275)
DEFECT SIZE	none (p = 0.57)	none (p = 0.52)	none (p = 0.59)
DEFECT LOCALIZATION	none (p = 0.41)	none (p = 0.71)	none (p = 0.605)
DURATION OF SURGERY (DOS)	none (p = 0.27)	none (p = 0.06)	**yes** (p = 0.034)
COMPLICATIONS	———-	**yes** (p = 0.005)	**yes** (p = 0.005)
COMORBIDITIES	none (p = 0.97)	**yes** (p = 0.022)	none (p = 0.066)
PREOPERATIVE RADIOTHERAPY	none (p = 0.795)	**yes** (p = 0.006)	none (p = 0.241)

## Discussion

A comparison with recent literature shows that our study is at the upper end of the range in terms of the study duration and number of cases. The period of data collection in other studies varied between 2 and 15 years, and the number of patients between 8 and 71.^4^, ^5^, ^6^, ^7^, ^8^, ^9^

Most studies are limited to the application of only a specific surgical technique or to the treatment of only a specific defect entity. Methodologically, the focus was often only on the analysis of the defect size, age, and gender.^5^^,^^6^^,^^10^^,^^11^

Concerning the distribution of entities, it was evident that our experience is in agreement with those in earlier studies.^4^^,^^6^^,^^8^^,^^9^^,^^12^ Often only defects concerning skin tumor excisions were analyzed.^13^^,^^14^ In our study, however, we often observed secondary, postoperative defects after neurosurgical interventions due to a malignant intracranial disease.^15^ Cranioplasty was frequently performed during these operations. Numerous studies associate them with typical complications, 2 of which occur most frequently, i.e., wound infection and exposed implants due to insufficient wound healing.^16^^,^^17^

We did not find any recent studies explicitly investigating the relationship between different defect etiologies and HS or POC. In our study, we investigated these factors, but we did not find any significant correlation. The large difference between the shortest and longest HS can be attributed to the fact that scalp defects often occurred during the treatment of a defined main diagnosis and were merely co-treated, e.g., as part of a neurosurgical therapy. The HS was then usually determined by the duration of treatment of the main disease. Therefore, some patients were discharged considerably later, even after the treatment of a scalp defect was completed.

Local flaps are the main technique used to treat scalp defects. They offer well-vascularized, long-lasting, and sometimes hair-bearing tissue. They are commonly used for moderately large wounds that are not suitable for primary wound closure or free flap.^12^^,^^18^ However, their use is limited by the defect size. The literature provides conflicting information regarding the maximum defect size that can be covered using local or regional flaps, with the defect size ranging from 10 to 150 cm².^9^^,^^12^^,^^19^^,^^20^ In our study, the average defect size was 25.6 cm² (1.2–78.5 cm²), which is within the range described in the literature. In the case of smaller defects ranging from 1 to 3 cm², direct wound closure can be discussed. However, owing to the firmness of the scalp, applicability of direct suture is limited. In most cases, generous subaponeurotic undermining and galeotomies are necessary to achieve tension-free approximate wound margins.^21^^,^^22^

We found that there was no significantly higher complication rate after flap reconstruction in a particular scalp area. Furthermore, there were no differences in HS and POC. Local flaps were successfully used to close defects in all scalp regions. Our overall experience is consistent with those reported in the literature. A major advantage of local flaps is the ability to close defects in a “like with like” manner. However, the precise knowledge of the anatomical details, biomechanics of the skin, physiology of the hair, and the presence of sufficient healthy donor tissue is essential.^12^^,^^18^^,^^19^^,^^23^ A wide variety of different flap designs that consider blood supply or use of tissue expanders extend the available options. In most cases, they enable a cosmetically appealing reconstruction with low donor-site morbidity.^7^^,^^24^

The mean DOS for the patients in this study was 125.4 ± 54.9 min (30–276 min). This wide range can be explained by the fact that several patients underwent neurosurgical treatment in the same session, which extended the DOS. Interestingly, we observed that there was no significant difference in DOS between patients with and without complications. Thus, DOS does not appear to be a risk factor for complications. In the literature, studies on risk factors report contrasting results, e.g., prolonged surgery can cause postoperative events such as local infections. Extending the surgery by 30 min can increase the mortality by 17% in individuals aged ≥80 years.^25^^,^^26^

Few data have been published as the comparison of DOS between different surgical techniques.^3^^,^^9^ However, some reports indicate that in local or regional flaps, the surgery is shorter and postoperative patient morbidity is lower than in free flaps.^8^^,^^27^

Our complication rate is comparable with the experience from other clinical centers, where a rate of 16.4% to 30% for local complications and 10% to 32% for revisions has been reported.^4^, ^5^, ^6^^,^^12^^,^^28^, ^29^, ^30^

We investigated the different defect etiologies, comorbidities, and neoadjuvant irradiation with regard to a possible association with complications. A significant correlation could not be confirmed, even though 50% of the patients with revisions had been irradiated earlier. Some contradictory statements are described in the literature on this aspect. Newman et al. could not identify a single pre-existing condition as an independent risk factor for postoperative complications, whereas Mueller et al. reported that wound healing can be compromised by comorbidities such as manifest diabetes or excessive smoking. Lee and Thiele reported that healing of surgical wounds is negatively correlated with preoperative radiotherapy. Some authors support this conclusion, while others offer contradictory statements. This illustrates that this topic is still controversial. Our study is biased by the relatively short follow-up periods. We can only hypothesize that a longer postoperative observation far beyond the POC would provide evidence on a positive correlation between radiotherapy and local complications, and thus worsen our long-term outcomes. However, previous scalp surgeries were not an exclusion criterion for flap surgery in most of our patients. To minimize the risk of complications, among other things, we shave the entire scalp to exactly assess the course of any scarring and thus plan the current flap more accurately. Considering this reduces the risk of circulatory problems and subsequent complications. If the course of the scar does not permit a local flap, we plan alternatives such as a free flap. Therefore, all the aforementioned factors should be considered before surgery.^9^^,^^27^^,^^31^, ^32^, ^33^, ^34^, ^35^

Our results show that neither HS nor POC depended on the surgical technique, defect etiology, size, and location. The DOS showed no correlation with HS, but a significant correlation with POC. The statistical analysis revealed a significantly longer HS of patients with one or more comorbidities and of patients with a history of irradiation. Interestingly, patients with pre-existing conditions or after radiotherapy did not have a longer POC. In case of complications, patients had to stay in hospital significantly longer, even postoperatively. A comparison of these aspects with the data from the literature was not possible because we could not identify any study concerning scalp reconstructions that explicitly examined the relationship between HS or POC and the factors that we were investigating. Notably, our patients had an average HS of 13.1 days, which was longer than those in other clinical centers.^8^ Therefore, it is important to differentiate whether the patient primarily comes for treatment of a scalp defect or whether the defect occurred secondarily while treating another main entity, such as, a neurosurgical disease.

Patient compliance is an important factor as well. In one case, extensive epitheliolysis developed after the patient was exposed to sub-zero temperatures after leaving the ward. Local hypothermia has a negative effect on wound healing due to the resulting vasoconstriction. Clinical studies have demonstrated that postoperatively, especially after flap reconstructions, the transplanted tissue appears to be at risk from the toxic effects of smoking. Necrosis rates increase with the number of cigarettes smoked per day, and the harmful mechanisms are multifactorial.^36^^,^^37^

### Limitations

One of the drawbacks of our study is the lack of a direct comparison with other conservative and surgical modalities.^3^ Free flaps are described as the gold standard in the treatment of medium and large defects.^12^^,^^19^ Complex injuries, such as those with exposed skull bone, pre-irradiation, implants, or chronic infections, can be treated in this way.^38^

**Figure 2:**
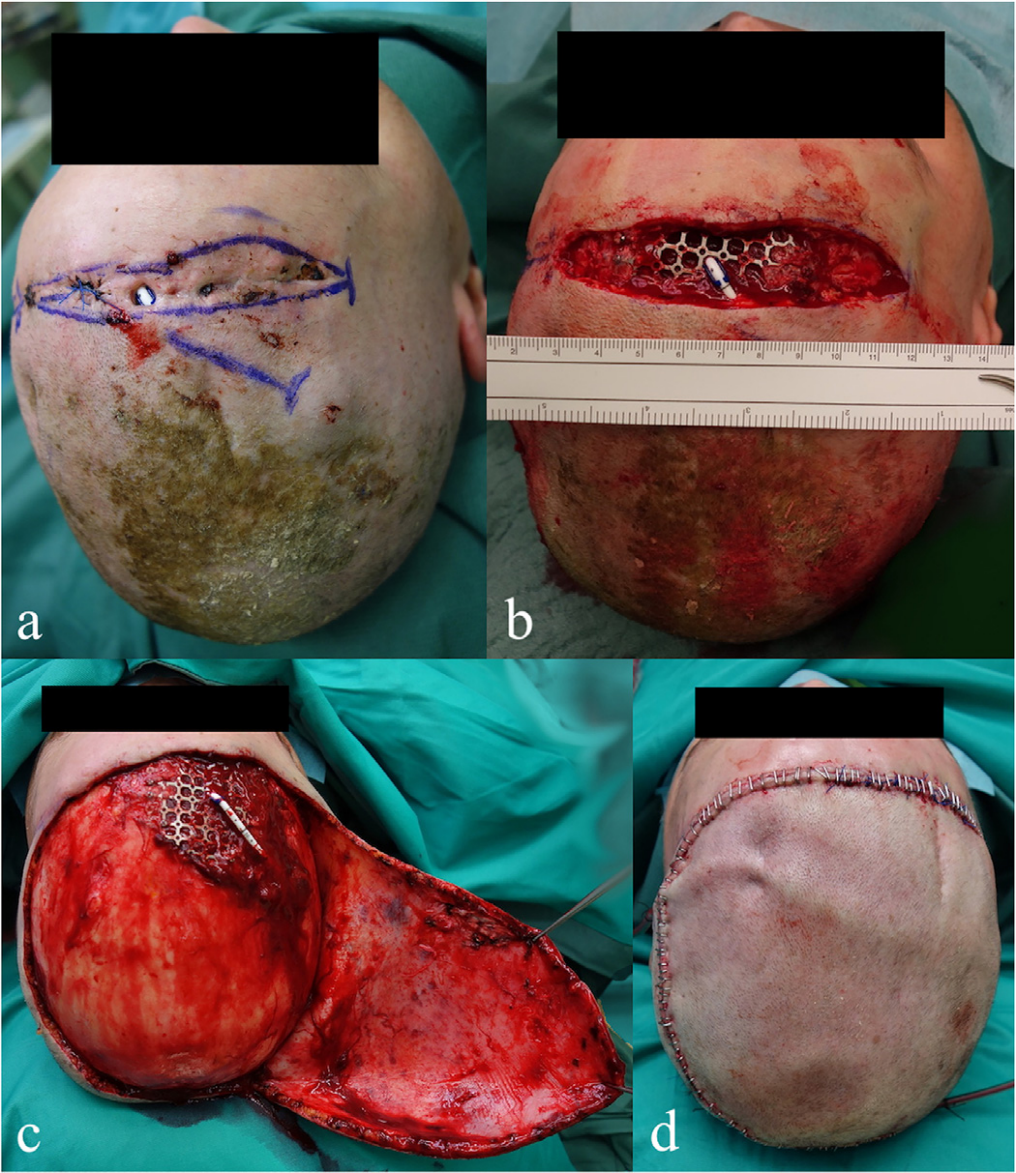
Difference between the initial defect size (a) and after a radical debridement (b); flap dimensions required for reconstruction (c) and final result (d).

In our clinic, we rarely have an indication to perform reconstruction using free flaps. Consecutively, a multicenter analysis to enroll a sufficient number of cases for a comparison with other techniques would be beneficial. Another limiting factor was the large number of defect sizes that could not be determined. In several cases, no information on the dimensions of the defects was provided in the charts. The photographs were not always taken as they were not mandatory. In this context, it would be advisable to create a standard for written and photographic documentation. Intraoperative photographs, with a ruler applied for scale, of the defects directly after definitive debridement may be beneficial. As it could document the actual dimensions of the defect to be reconstructed (Figure 2).

## Summary

Our data suggest that local and regional flaps could be safe and reliable options for the treatment of scalp defects up to 80 cm² in carefully selected patients. Furthermore, our results indicate that postoperative complications do not appear to be related to defect etiology, surgical technique, defect size, defect localization, surgery duration, comorbidities, and radiotherapy. Such complications, comorbidities, and neoadjuvant radiotherapy have an impact on the length of hospitalization. The postoperative inpatient course is only influenced by the duration of the surgery and possible postoperative complications. Defect etiology, surgical technique, defect size, and defect localization have no apparent impact on the overall hospitalization.

## Conflict of interest

All authors declare, that they have no conflict of interest.
